# Cysteine and homocysteine as biomarker of various diseases

**DOI:** 10.1002/fsn3.1818

**Published:** 2020-08-12

**Authors:** Tahniat Rehman, Muhammad Asim Shabbir, Muhammad Inam‐Ur‐Raheem, Muhammad Faisal Manzoor, Nazir Ahmad, Zhi‐Wei Liu, Muhammad Haseeb Ahmad, Azhari Siddeeg, Muhammad Abid, Rana Muhammad Aadil

**Affiliations:** ^1^ National Institute of Food Science and Technology University of Agriculture Faisalabad Pakistan; ^2^ School of Food Science and Engineering South China University of Technology Guangzhou China; ^3^ Institute of Home and Food Sciences Government College University Faisalabad Pakistan; ^4^ College of Food Science and Technology Hunan Agricultural University Changsha China; ^5^ Department of Food Engineering and Technology Faculty of Engineering and Technology University Gezira Wad Medani Sudan; ^6^ Institute of Food and Nutritional Sciences Pir Mehr Ali Shah Arid Agriculture University Rawalpindi Pakistan

**Keywords:** biomarker role, CVD, cysteine, diabetes, homocysteine, neurological disorders, vitiligo

## Abstract

Cysteine and homocysteine (Hcy), both sulfur‐containing amino acids (AAs), produced from methionine another sulfur‐containing amino acid, which is converted to Hcy and further converted to cysteine. This article aims to highlight the link between cysteine and Hcy, and their mechanisms, important functions, play in the body and their role as a biomarker for various types of diseases. So that using cysteine and Hcy as a biomarker, we can prevent and diagnose many diseases. This review concluded that hyperhomocysteinemia (elevated levels of homocysteine) is considered as toxic for cells and is associated with different health problems. Hyperhomocysteinemia and low levels of cysteine associated with various diseases like cardiovascular diseases (CVD), ischemic stroke, neurological disorders, diabetes, cancer like lung and colorectal cancer, renal dysfunction‐linked conditions, and vitiligo.

## Graphical abstract

Cysteine and homocysteine, both sulfur‐containing amino acids (AAs) produced from methionine another sulfur‐containing amino acid, which is converted to homocysteine and further converted to cysteine. The aim of this article is to highlight the link between cysteine and homocysteine, and their mechanisms, important functions, play in the body and their role as a biomarker for various different types of diseases. Hyperhomocysteinemia (elevated levels of homocysteine) is considered as toxic for cells and is associated with different health problems. Not only hyperhomocysteinemia but also low levels of cysteine in most cases is used as a biomarker for various diseases like cardiovascular diseases (CVD), ischemic stroke, neurological disorders, diabetes, cancer like lung and colorectal cancer, renal dysfunction‐linked conditions, and vitiligo.

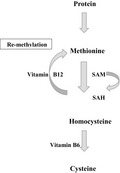

## 1 INTRODUCTION

Cysteine (Cys) the primary sulfur‐containing amino acid (SAA) is a semiessential amino acid (AA) because it can be obtained from the diet or produced from methionine degradation via the transsulfuration pathway. In the mammalian diet, cysteine is considered as representative of SAAs (Bin, Huang, & Zhou, 2017). Cysteine belongs to a group of amino acids (AAs) which contain polar and uncharged R group which is more hydrophilic than AAs bearing nonpolar side chain. Cysteine undergoes oxidation at thiol group (–SH) which has the ability to form a covalent bond by reacting with free radicals and other groups, for example, cysteine linked by disulfur bridge. This bridge is stronger than hydrogen bonds (H–bonds), Van der Waals forces, and salt bridge (bond between electrically charged acidic and basic groups, especially on a protein) but weaker than peptide bonds. The most abundant form of cysteine in our body is L‐cysteine. Cysteine is synthesized in our body from methionine (sulfur‐containing essential amino acid) which is abundant in cheese, yogurt, meat, chicken, turkey, wheat gums, beef, and nuts (Sameem, Khan, & Niaz, 2019).

Homocysteine (Hcy) is also a sulfur‐containing amino acid‐like cysteine and methionine. Hcy is an essential AAS with a molar mass of 135.18 g/mol and formed during the conversion of methionine to cysteine. In humans, the only pathway for the biosynthesis of Hcy is from methionine (Ntaios, 2015). Hcy was discovered in 1932 by Butz and du Vigneaud when they heated methionine in sulfuric acid and obtained a substance with features similar to cysteine and named it “homocysteine (Hcy)” because it was a homolog of cysteine (Tsiami & Obersby, 2017). Hcy obtained through the methionine cycle as an intermediate product is catabolized through the transsulfuration pathway into cysteine (Ostrakhovitch & Tab ibzadeh, 2015). Hcy exists in protein‐bound Hcy and free Hcy forms, and some of these two are referred to as total Hcy (tHcy). Hcy cannot be obtained from the diet since it is produced in the body from methionine which acts as a precursor of Hcy (Tsiami & Obersby, 2017).

### 1.1 Metabolism of cysteine and HCY

Within the body, cysteine is synthesized in the liver from Hcy by transmethylation of methionine. First, Hcy is condensed with serine by cystathionine β‐synthase (CBS) and then cleavage of CBS produces cysteine. During transsulfuration, serine gives its carbon chain to cysteine and sulfur atom of cysteine comes from methionine. Within the body, cysteine catabolic pathways are sources of the synthesis of coenzyme A, glutathione, taurine, and oxidized and reduced inorganic sulfur. In the liver, two catabolic pathways of cysteine take place which includes oxidative pathway and desulfuration pathway, respectively. Briefly, in the oxidative pathway, cysteine sulfinate (intermediate in cysteine metabolism) is either transaminated to produce sulfite and pyruvate or decarboxylated to form taurine. The desulfuration pathway ends up with hydrogen sulfide and pyruvate. If the supply of cysteine is high, then the oxidative pathway is superior over desulfuration pathway and the desulfuration pathway increases when cysteine supply is low (Papet et al., 2019).

Metabolism of Hcy involves two pathways, which mainly include remethylation and transsulphuration. Remethylation is the process that requires methyl group for the conversion of Hcy into methionine, and the Remethylation process is carried out by betaine–homocysteine methyltransferase (BHMT) in the kidney and liver. Transculturation involves attachment of Hcy with serine and formation of cystathionine (a sulfur metabolite produced from Hcy) with the help of CBS (an enzyme) and vitamin B6 which acts as a coenzyme to synthesize cysteine (Hannibal & Blom, 2017; Ntaios, 2015). Cystathionine is hydrolyzed by an enzyme cystathionine γ‐lyase (CL) and forms α‐ketobutyrate and cysteine. Remethylation of Hcy can occur through the folate cycle in which it is catalyzed by vitamin B12 in the presence of an enzyme called methionine synthase to be recycled into methionine. The above two pathways are controlled by S‐adenosylmethionine (SAM) which acts as an activator of CBS. If the diet is rich in methionine, then the conversion of dietary methionine into SAM occurs, and as a result, CBS activation increases, and transculturation is dominated over remethylation. On the other hand, if the diet is low in methionine then SAM concentration is not enough for activation of CBS and the result is remethylation of Hcy promoted over transsulfuration (Tsiami & Obersby, 2017).

### 1.2 Circulation of HCY

Homocysteine (Hcy) is metabolized in kidneys and liver, whereas in the pancreas and small intestine transsulfuration takes place. In the human body after the production of the low level of Hcy, almost 3% circulate freely in the body, with the majority of Hcy present in bound form with other molecules or in disulfide form (Rizzo & Sciorsci, 2018). Total plasma homocysteine (tHcy) is the sum of the circulating Hcy molecules either in its reduced or oxidized forms. The majority of tHcy about 98%–99% in its disulfide form is oxidized rapidly by reacting with other molecules that contain free thiol group like albumin (protein‐containing free cysteine) and remaining exists as reduced form. Circulation of Hcy in our body is regulated by transsulfuration and remethylation pathways discussed above and by reabsorption in the kidney (Barroso, Handy, & Castro, 2017).

### 1.3 Normal concentration in the body

In human plasma, Hcy concentration is typically below 12–15 μM and the cysteine concentration level is 240–360 μM (Wang et al., 2019). Hcy level is high in males as compared to females; it may be due to gender differences in Hcy metabolism and low concentration of vitamin B12 and folate in males. According to a population‐based cross‐sectional study, average Hcy concentrations were 12.6 in men and 9.6 μmol/L in women, respectively, and increase with age like 4.6–8.1 µmol/L in age 0–30 years, 6.3–11.2 µmol/L at age of 30–59 years in males and 4–5–7.9 µmol/L in females, and 5.8–11.9 µmol/L for age above 59 years (Cohen, Margalit, Shochat, Goldberg, & Krause, 2019).

## 2 ROLE OF CYSTEINE IN THE BODY

Cysteine is known as a proteinogenic amino acid because it acts as a building block about 2% of proteins and plays an important role in biological processes carried out in our body. It catalyses many important metabolic reactions (Sameem et al., 2019). It takes part in lipid biosynthesis (Sameem et al., 2019), iron–sulfur biosynthesis (Bak, Bechtel, Falco, & Weerapana, 2019), which is an important constituent of skeletal muscles (Papet et al., 2019), keratin (Wang, Yang, McKittrick, & Meyers, 2016), and is also a source of taurine, glutathione, and coenzyme A (Bin et al., 2017; Papet et al., 2019).

### 2.1 Lipid biosynthesis

Cysteine plays a vital role in the synthesis of essential fatty acids, and therefore, it is important for cell membrane and nerve myelin sheaths especially on axon endings (Sameem et al., 2019). Through shielding axons from oxidative stress, cysteine can prevent major neurodegenerative disorders such as Parkinson's or Alzheimer's and underlying stress (Hasanbasic, Jahic, Karahmet, Sejranic, & Prnjavorac, 2016). Moreover, dietary L‐cysteine supplementation can improve the lipid metabolism (Yin et al., 2016).

### 2.2 Skeletal muscles

Free sulfur AAs especially cysteine and methionine are an important part of skeletal muscles. However, their concentration as protein constituent is lower in skeletal muscles than in tissues. Both these thiol AAs are strictly required by skeletal muscles. Almost 1.4 g/16 g N cysteine contains skeletal muscles in mammals (Papet et al., 2019). Concentrations of different AAs in skeletal muscles are listed below in Table 1.

**Table 1:** The concentration of amino acids (AAs) in skeletal muscles

Amino acids	Concentration in skeletal muscles	Reference
Cysteine	19 μmol/g	
Methionine	32 μmol/g	(Papet et al., 2019)
Taurine	9 to 19 μmol/g	
Glutathione	2.3 μmol/g	

### 2.3 Cysteine in cellular organelles

In proteins, cysteine residues play many functional roles. Sulfur atom in cysteine which is oxidative sensitive and highly ionizable is responsive to redox conditions and pH of subcellular organelles in eukaryotes. Its function is highlighted in mitochondria and endoplasmic reticulum (ER) especially. Mitochondria are known as the powerhouse of the cell, involved in the oxidation of fatty acids, breakdown of AAs, aerobic metabolism, and phosphorylation. Cysteine residues are important for many mitochondria‐associated unique functions like iron–sulfur biosynthesis and help in the electron transport chain by providing redox cofactors. Cysteine residues support many processes in ER; especially synthesis, trafficking and folding of proteins, and also lipid biosynthesis, detoxification, and steroid metabolism carried out in ER (Bak et al., 2019).

### 2.4 Keratin

Invertebrates, keratin is a very important structural protein and is an essential part of nail, hair, and exoskeleton in many species (Paul, Sbodio, & Snyder, 2018). Cysteine is the main part of keratin because it has a large number of cysteine residues having a thiol group that provides strong covalent disulfide bonds and due to these linkages keratin has insoluble property. Based on sulfur, keratin can be classified as soft and hard keratin. Softer keratin has less sulfur and is abundant in the epidermis (outermost layer of the skin), and harder keratin has a higher amount of sulfur in it (hair, nails) (Wang et al., 2016). The percentage of cysteine in skin and hair is almost 10%–14% (Paul et al., 2018).

### 2.5 Sources of Taurine, Glutathione, and Coenzyme A

Taurine plays important physiological functions like anti‐inflammatory, neural and visual development, and detoxification in our body (Inam‐u‐llah et al., 2018). In mammals, taurine is the most abundant free AAs and is produced during the metabolism of cysteine. Many types of research show that taurine level increases by increasing cysteine supplementation in HIV‐infected patients. Glutathione is cysteine‐containing tripeptide and synthesized from cysteine. It plays a vital role in cellular antioxidation and acts as antilipid peroxidation (Bin et al., 2017). Cysteine is also involved in the synthesis of coenzyme A which plays important role in cellular oxidative pathways like Kreb's cycle, amino acid oxidation, fatty acid β‐oxidation, and also in protein modification and lipid synthesis (Papet et al., 2019).

### 2.6 Supplementation of cysteine

N‐acetyl cysteine (NAC) is a precursor of cysteine and normally given as a cysteine supplement; it protects the cell from different types of drugs and chemicals. It not only plays an important role in decreasing the risk of cancer, diabetes, respiratory system‐related problems, flu, and influenza but also helps in improving fertility by decreasing ROS (reactive oxygen species) level and increasing sperm motility and count. Some studies also have shown decreased osteoporosis linked with cysteine supplementation (Sameem et al., 2019). Briefly explain the results of some studies in Table 2.

**Table 2:** Other roles of cysteine

Other roles of cysteine in the body	Reference
Promotes muscle function	(Paul et al., 2018)
Prevents neurodegenerative diseases by preventing axon from environmental and oxidative stress	(Sameem et al., 2019)
Boosts the immune system	(Sameem et al., 2019)
Enzymatic production of hydrogen sulfide (H2S)	(Kohl, Mellis, & Schwarz, 2019)
Cys positively correlates with bone mineral density and fat mass	(Elshorbagy et al., 2009)

## 3 HOMOCYSTEINE (HCY) ROLE IN THE BODY

Homocysteine (Hcy) is produced from methionine as an intermediate product which is converted into cysteine and remethylated into methionine as discussed above. The major role of Hcy in our body is it acts as a biochemical intersection between methionine metabolism and biosynthesis of cysteine which plays various important roles in our body (Mishra, 2016). Hcy can be used in one of two ways. These ways may include remethylation of Hcy into methionine in case of methionine deficiency, and when methionine is an insufficient amount, Hcy can be used to produce cysteine in presence of vitamin B6 (Ganguly & Alam, 2015). Almost 50% of intracellular Hcy is used in the production of cysteine (E. A. Ostrakhovitch & Tabibzadeh, 2015). Hcy elevated level in the body can cause various diseases like kidney dysfunction (Barroso et al., 2017), CVD, ischemic stroke (Ntaios, 2015), and neurological problems like autism (Żurawicz & Kałużna‐Czaplińska, 2015). Some studies on rats indicated that high tHcy caused Parkinson's disease, Alzheimer's disease, and seizures (Lehotský et al., 2016). Hcy may play a role in oxidative stress, enhancing the production of reactive oxygen species, and hence may cause lipid peroxidation and cell membrane injury (Sławek & Białecka, 2015). Low Hcy level is associated with vitamin B12, vitamin B6, and folate‐rich diet especially fruits and vegetables or their supplementation (Ntaios, 2015). Elevated Hcy is also associated with increased homocysteine thiolactone (HCTL) which is a highly reactive molecule and responsible for alternation of biological activity and physicochemical properties of proteins (Bhargava, 2018).

### 3.1 Hyperhomocysteinemia

Abnormal elevation in the level of Hcy in the plasma (above 15 µmol/L) is referred to as hyperhomocysteinemia (HHcy). Elevated total plasma homocysteine tHcy is considered toxic for cells and is associated with different health problems. Many genetic defects in Hcy pathways which are discussed above and vitamin B12 and folate deficiencies lead to increased cellular Hcy level. Severe hyperhomocysteinemia leads to a condition called homocystinuria (elevation in tHcy level due to abnormal methionine metabolism). It is very necessary to export elevated Hcy from the intracellular environment into the systemic circulation. The liver and kidneys are responsible for its clearance because of BHMT and CBS, which convert Hcy into nontoxic metabolites (Hannibal & Blom, 2017). In a healthy human, the Hcy level in plasma is from 5 to 15 µmol/L and the optimum level of tHcy is suggested to be <10 μmol/L (Tsiami & Obersby, 2017). According to Hcy level in plasma, HHcy is classified according to different levels: severe (>100 µmol/L), intermediate (31–100 µmol/L), and moderate (16–30 µmol/L) (Kostić et al., 2019).

### 3.2 CAUSES OF HYPERHOMOCYSTEINEMIA

Circulation of Hcy depends upon many factors which include genetic and nongenetic factors. In nongenetic factors, vitamin B12, vitamin B6, cofactors for Hcy regulatory enzymes, and folate have a great influence on the concentration of Hcy in plasma. An inverse relationship has been reported between the concentration of Hcy and these factors in the human body. Apart from that, kidney dysfunction is also linked with hyperhomocysteinemia. Mild HHcy can be a result of our lifestyle modifications like smoking, alcohol consumption, lack of exercise, tobacco, caffeine used, and aging. Genetic factors include a mutation in pathways that are involved in Hcy metabolism (Barroso et al., 2017). Gender differences are also known to affect Hcy levels with high concentrations of Hcy being observed in males as compared to females. The study showed 15.1% HHcy in males and 3.4% in females below 55 years of age and in above 55 years 16.6%, and 5.8% were recorded in males and females, respectively (Cohen et al., 2019).

### 3.3 Role of nutritional cofactors in HHCY

Nutritional cofactors like folate, vitamin B6, and vitamin B12 have a great influence on HHcy because these cofactors are vital in the metabolism of Hcy. Deficiency in their level leads to elevated tHcy, and by increasing the level of these cofactors, we prevent HHcy. According to a meta‐analysis, folic acid supplementation (0.5–5 mg daily) results in a reduction of tHcy by about 25%. Vitamin B12 (0.5% additional) reduces Hcy concentrations by another 7% (Mishra, 2016). Another study showed that supplementation of vitamin B12 and folate reduced Hcy levels by 7% and 23%, respectively (Cheng et al., 2016). A 12%, 5%, and 43% reduction in Hcy level was seen with vitamin B6, vitamin B12, and folate, respectively (Ntaios, 2015). Table 3 shows some of the common sources of folate, vitamin B12, and vitamin B6.

**Table 3:** The Sources of folate, vitamin B6, and vitamin B12

Vitamins	Sources	References
Vitamin B12	Egg, cheese, vegetables, fish, milk, and fortified foods	(Moll & Davis, 2017)
Vitamin B6	Meat, fish, green leafy vegetables, legumes, egg, potato, and milk	(Mishra, 2016)
Folate	Lentils, orange juice (raw), and vegetables	(Looman et al., 2018)

## 4 CYSTEINE AND HOMOCYSTEINE AS BIOMARKER OF VARIOUS DISEASES

### 4.1 Cardiovascular diseases

In developing and developed countries, cardiovascular disease (CVD) is the leading cause of death worldwide. Many studies relate to elevated Hcy role in the development of various forms of vascular diseases (Hannibal & Blom, 2017). According to some researchers, the concentration of plasma Hcy above 10 µmol/L is associated as a risk factor in the development of CVD and ischemic heart disease (Ostrakhovitch & Tab ibzadeh, 2019). A meta‐analysis indicated that a 25% elevated plasma Hcy level was associated with 10% and 20% increased risk of CVD and stroke, respectively. Another meta‐analysis showed that when serum Hcy level decreased by 3µmol/L, a 16% reduction in coronary heart disease was seen. A 5 µmol/L increase in plasma increased the relative risk of coronary heart disease by 1.6–1.8 times (Ntaios, 2015). Another meta‐analysis showed that for each 5 μmol/L increase in Hcy, the risk of mortality increased by 32%, and the risk of heart disease increased by 52% (Shiao, Lie, & Yu, 2018). Hcy may cause CVD through various mechanisms such as the increased proliferation of muscle cells that cause narrowing of vessels, alter blood coagulant properties, cause oxidant injury to the vascular endothelium, and damage arterial walls (Mishra, 2016). Table 4 summarizes the effects of hyper Hcy on cardiac health.

**Table 4:** The effects of hyperhomocysteine on CVD

Study design	Result	References
Obese and overweight individuals, divided into two groups with metabolic syndrome or without	The relation between Hcy and hypertension and hyperlipoproteinemia showed Hcy as a biomarker of CVD	(Sreckovic et al., 2017)
Assessing the severity of CAD by obtaining and analyzing the data from 70 participants undergoing coronary angiography at Kasturba Hospital, Manipal University	Serum Hcy is an independent risk factor for coronary artery disease	(Shenoy, Mehendale, Prabhu, Shetty, & Rao, 2014)
A total of 30 healthy individuals and 30 renal transplant recipients studied for about 3 months to determine the Hcy role in the occurrence of CVD	Plasma Hcy failed to show as an independent risk factor for a cardiovascular event in patients with successful renal transplant	(Goswami et al., 2018)
A total of 100 overweight women divided into a placebo group and vitamin D3 receiving group for 2 months, and Hcy, calcium, and BMI measured.	Vitamin D3 receiving group showed decreased Hcy level which in turn minimized the risk of CVD	(Al‐Bayyari, Al‐Zeidaneen, & Hamadneh, 2018)
Epidemiological studies of more than 10,000 patients	Elevated Hcy may be a strong predictor of cardiovascular mortality	(Mishra, 2016)
Lipid profile, Hcy, vitamin B, B12, and folate measured in the control group and patients with nonalcoholic steatohepatitis	Nonalcoholic steatohepatitis associated with elevated Hcy levels is an independent cardiovascular risk factor	(Leach et al., 2014)
Two cohort studies of women in the Nurses’ Health Study and men in the Health Professionals Follow‐up Study	Plasma Hcy was directly associated with PAD (peripheral artery disease) in men as compared to women	(Bertoia et al., 2014)
Review on eleven meta‐analyses and two systemic review and other studies	Increased Hcy level can be a risk factor for primary CVD, and according to certain studies, Hcy reduced CVD events. tHcy can be employed as an independent biomarker	(Obersby, Chappell, & Tsiami, 2013)
7,591 men and 8,585 women, 40 to 67 years of age	Elevated plasma Hcy level was associated with major components of cardiovascular risk profile, ie, sex, age, smoking, high blood pressure, elevated cholesterol level	(Mishra, 2016)
A cross‐sectional study on 234 children and adolescent	Children with parental history of CVD had higher serum Hcy levels than those without such history	(Leal et al., 2013)

Hcy was positively associated with both diastolic and systolic blood pressures; for instance, when Hcy concentration increased by 5 μmol/L, diastolic and systolic blood pressure in men increased by 0.5 and 0.7 mmHg, respectively, and in case of women, Hcy and blood pressure showed stronger correlation and increased by 0.7 and 1.2 mmHg (diastolic and systolic) (Ganguly & Alam, 2015).

Epidemiological research demonstrated a U‐shaped relationship between cardiovascular diseases and tCys after adjustment of other risk factors and Hcy (De Chiara et al., 2012). Van den Branbdhof and his colleagues found no relationship between tCys and the risk of coronary heart disease. A study was conducted to investigate the relationship between tCys and risk of mortality and CVD hospitalizations on men and women in the Hordaland Hcy Study cohort and concluded that tCys was not associated with all cause of CVD or non‐CVD mortality (van den Brandhof, Haks, Schouten, & Verhoef, 2001). A study was conducted, later on, to determine the relationship between tHcy and CVD and non‐CVD‐related mortality and morbidity, and results demonstrated that tHcy as a biomarker of CVD and non‐CVD mortality and morbidity (El‐Khairy, Vollset, Refsum, & Ueland, 2003).

### 4.2 Ischemic stroke

In order to determine the association between elevated tHcy level in acute stroke with mortality, an experiment was conducted to analyze whether during the convalescent phase; after an acute ischemic stroke, change in Hcy level in plasma contributed to the risk of ischemic stroke. The study showed that during the convalescent phase, elevated Hcy was independently associated with an increased risk of recurrent ischemic stroke after the index cerebrovascular event. So, researcher concluded that tHcy used as the biomarker of ischemic stroke during the convalescent phase of stroke (Shi et al., 2018). Another study showed that 5 µmol/L increase in tHcy elevates the relative risk of ischemic stroke by 1.5 times. When serum Hcy decreased 3µmol/L; a 24% reduction in ischemic stroke was seen (Ntaios, 2015).

### 4.3 Neurological disorders

Hcy is transported in the brain which has a limited capacity for Hcy metabolism. Brain tissues have different mechanisms to lower down and maintain Hcy level such as efficient recycling through vitamin B12‐dependent methionine synthase (only enzyme in the brain involved in Hcy conversion into methionine), catabolism through cystathionine beta‐synthase to cystathionine (a non‐noxious product which converts further into cysteine), and export to external circulation (Ganguly & Alam, 2015).

In the brain, accumulation of Hcy was associated with increased tHcy and SAM in cerebrospinal fluid. Induced Hcy caused endothelial and astrocytic dysfunction which resulted in altered neuronal function. Elevated levels of Hcy led to an enhanced excitatory glutamatergic neurotransmission in different brain regions which caused neuronal damage. In short, Hcy caused redox imbalance and increased oxidative stress and production of free radicals in many cells including endothelial, glial, and neuronal cells and led to several neurological disorders (Lehotský et al., 2016). On the other hand, cysteine plays an important role in redox homeostasis. Redox‐modulated events play important roles not only in peripheral tissues but also in the brain where cysteine disposition is central to these pathways. Disrupted redox homeostasis played an important role in disease progression and neurodegenerative disorders, and Cys, being an antioxidant, is responsible for neutralizing much of the oxidative damage generated (Paul et al., 2018).

### 4.4 Autism spectrum disorder

Nowadays autism spectrum disorder (ASD) is the topic of many types of research with AAs being used as a biomarker of ASD. ASD is the neurodevelopmental disorder that occurs early in life and is associated with abnormal functioning of CNS. ASD is estimated to be prevalent in almost 11.3 per 1,000 children age 8 years. Besides cysteine and Hcy, other AAs like tyrosine, threonine, methionine, phenylalanine, and other brain AAs also considered as a biomarker of ASD (Żurawicz & Kałużna‐Czaplińska, 2015). Studies also reported less concentration of Cys in ASD patients and control after overnight fasting. These findings are compatible with increased oxidative stress and decreased detoxification capacity in ASD (ElBaz, Zaki, Youssef, ElDorry, & Elalfy, 2014; Geier et al., 2009). Similarly, another study reported low intracellular cysteine concentration in children suffering from ASD as compared to the healthy group and observed elevated levels of Hcy in the peripheral blood mononuclear cells (Suh, Walsh, McGinnis, Lewis, & Ames, 2008). Tu, Chen, and He, (2012) observed a high concentration of Hcy in the fasting plasma of ASD patients. Moreover, a high level of Hcy in plasma and urine may due to vitamin B deficiency. James et al. (2004) observed a low level of Hcy in fasting plasma, this difference may due to different ages of participants, and James et al also observed low level of cysteine in the plasma of ASD children as compared to the control group. There are two studies by Kałużna‐Czaplińska, Żurawicz, Struck, and Markuszewski (2014), and Kałużna‐Czaplińska, Michalska, and Rynkowski (2011) also reported a high level of Hcy in the urine of ASD children than the healthy group.

### 4.5 Dementia

Dementia is not a specific disease but a clinical syndrome that includes different diseases like Parkinson's disease, Alzheimer's disease (AD), Lewy body diseases (LBD), and vascular dementia (VAD). A 50% increase in the risk of dementia was seen when 5‐µmol/L increase was observed in tHcy. Hcy may be harmful to nervous tissue due to various reasons since it causes increased stimulation of receptors, increased activation of pathological protein formation, and direct neurotoxicity (Sławek & Białecka, 2015).

### 4.6 Alzheimer's disease

The most common form of dementia is AD, and the risk of AD is almost double in people over 50 years of age with tHcy level higher than 14 μmol/L (Sławek & Białecka, 2015). The abnormal increase in tHcy can be used as a biomarker of AD. HHcy in AD may be due to folate or vitamin B12 deficiency. A study is conducted to explore the relationship between HHcy and vitamins as the disease progresses (Farina, Jernerén, Turner, Hart, & Tab et, 2017). A meta‐analysis showed that high tHcy and low folate and vitamin B12 concentrations may act as a risk factor of AD (Shen & Ji, 2015). Disrupted cysteine metabolism is seen in AD patients with some studies reporting an increased level of Cys in the AD patients. This could be attributed to impaired uptake of Cys into the cells, decreased level of H_2_S and disruption of Cys neuronal transporter in AD (Paul et al., 2018).

### 4.7 Epilepsy

For epilepsy (sudden occurrence of seizures in behavior), peripheral biomarkers are absent; notably, but in humans, few studies suggested these biomarkers. Redox changes were seen in animal models of epilepsies, and cysteine was considered as a biomarker of oxidative damage. The ratio of Cys/Cys (cysteine/cysteine) has been proposed to serve as a biomarker of redox status in plasma and tissues in humans and in animals. A study on rats concluded that a decrease in Cys and ratio of Cys/Cys in plasma could act as a redox biomarker in temporal lobe epilepsy (Liang & Patel, 2016). Hcy and its metabolites homocysteine thiolactone (HT) causes seizures by different unknown mechanisms. Elevated tHcy and HT levels have been identified as risk factors for the development of numerous brain disorders including epilepsy (Hrnčić et al., 2014).

### 4.8 Head injury

Head injury is among the numerous causes of mortality and morbidity in young people. Total plasma homocysteine is used as a biomarker of various neurological outcomes after head injury. An experiment showed that after a head injury, elevated tHcy has an independent impact on neurological outcomes. The study included patients (both males and females) who were admitted within 24 hr after head injury for 3 months, and their biochemical tests showed higher tHcy levels in severely injured patients and low tHcy in mildly injured patients. The elevated levels of tHcy after a head injury can be due to metabolic stress response similar to the results of subarachnoid hemorrhage (a type of stroke), caused by down regulation of cystathionine‐β‐synthase. The result showed a significant relationship between tHcy and neurological outcomes (Dhandapani et al., 2018).

### 4.9 Diabetes

Diabetes is the most common metabolic disorder associated with hyperglycemia. It is suggested that a high level of Hcy in diabetes is the biomarker of microvascular complications like diabetic neuropathy, retinopathy, and nephropathy. Various studies also suggest that in poorly controlled type‐2 diabetes mellitus increased level of Hcy is associated with increased risk of macrovascular complications like atherosclerosis and cardiovascular disease (Mundu, Kumar, Mitra, Kumar, & Sinha, 2017). HHcy in type 2 diabetes is associated with a high prevalence of nephropathy (Rujaswini, Praveen, Chowdary, Aanandhi, & Shanmugasundaram, 2018). Emerging evidence shows a positive role of L‐cysteine‐rich diet in several pathological conditions of type‐2 diabetes and neurodegenerative diseases (Yin et al., 2016). Different studies showed that in diabetic patients, cysteine homeostasis changed and resulted in lower cysteine blood levels. In type 2 diabetic patients, L‐cysteine (LC) supplementation lowered oxidative stress which was considered as a risk factor in the progression of vascular inflammation in diabetes (Manna & Jain, 2013). L‐cysteine can be strongly considered in antidiabetic drugs in the management of type 2 diabetes (Salman, Refaat, Selima, El Sarha, & Ismail, 2013).

### 4.10 Diabetic neuropathy

Diabetic neuropathy is the condition of diabetes in which nerve damage and dysfunction occur. The study indicated that Hcy is independently associated with the prevalence of diabetic neuropathy in collective type 2 diabetic patients (Rujaswini et al., 2018). Research is conducted to finding out the relationship between elevated Hcy level and microvascular complications in diabetics, and results showed that serum Hcy level in diabetes patients with microvascular complications is an important biomarker (Mundu et al., 2017).

### 4.11 Gestational diabetes mellitus

Gestational diabetes mellitus (GDM) is a metabolic complication with glucose intolerance during pregnancy. High plasma level of Hcy was found in pregnant women with GDM as compared to nonpregnant women with normal glucose tolerance (Gong et al., 2016). (Tarim et al., 2004) conducted a study to determine whether elevated plasma Hcy is associated with GDM in Turkish women and found that women with gestational diabetes with abnormal screening test results but normal oral glucose tolerance test results have higher Hcy levels than normal pregnant women. A cross‐sectional study was conducted, which include 223 pregnant women between 24 and 28 weeks of gestation. Sample divided into 3 groups based on their glucose tolerance, glucose intolerance, gestational diabetes, and normal controls, and serum Hcy level was prospectively measured. The result found an elevated level of Hcy during the second trimester among women with gestational diabetes mellitus as compared to normal controls (Guven, Kilinc, Batukan, Ekerbicer, & Aksu, 2006).

### 4.12 Renal dysfunction

Mild‐to‐moderate hyperhomocysteinemia is often associated with impaired kidney function. The majority of renal disease patients reported HHcy (Barroso et al., 2017). The kidney is the major site of Hcy metabolism, and any disturbance in Hcy metabolism pathways leads to an abnormal increase in its level which causes many renal problems and impairment in renal function. Renal dysfunction is responsible for further accumulation of Hcy, thus causing chronic renal failure. Studies showed that Hcy toxicity caused ischemic–reperfusion which led to kidney damage. Imbalanced homeostasis and increased oxidative stress and reactive oxygen species (ROS) which caused glomerular endothelial dysfunction also resulted in a change in glomerular filtration rate, which induced renal dysfunction as shown by increased Hcy and Cys. And Cys‐albumin increased in end‐stage kidney disease (Ostrakhovitch & Tabibzadeh, 2015). A study concluded that in nephropathic cystinosis, N‐acetyl‐cysteine (NAC) reduced oxidative stress and improved renal function (de Faria Guimaraes et al., 2014). Some recent studies demonstrated that NAC might prevent contrast nephropathy (impairment of renal function). It has anti‐inflammatory and antioxidant effects that could prevent renal dysfunction (Aldemir et al., 2016).

### 4.13 Renal cell carcinoma

In the United States almost 13,000 deaths and 50,000 new cases so that, RCC detection is increasing day by day. Kidney plays an important role in the regulation of plasma AAs and in the clearance of nitrogenous substances from our bodies. From glomerular filtration, renal tubule epithelial cells reabsorb AAs so that most tumors arise from renal tubule cells, and due to these tumors, change in the reabsorption of AAs is seen. In a study, a researcher obtained serum of 189 patients suffering from RCC at Fox Chase Cancer Center and stored at −70C. Serum samples of control groups from different sources like employees, spouses of patients, and other individuals at Fox Chase Cancer Center were collected. With the help of amino acid analyzer, their sample comparison showed alternations between 15 out of 26 AAs like taurine, threonine, glycine, alanine, serine, asparagine, glutamate, citrulline, methionine, tyrosine, ornithine, histidine, phenylalanine, and proline were decreased in RCC patients while cysteine and arginine were increased. This study shows that a significant difference between serum AAs may use for screening tests of RCC and may have potential clinical uses in the determination of RCC (Mustafa et al., 2011).

### 4.14 Cancer

Murphy et al. (2011) conducted a study that showed that high concentrations of cysteine were linked with low risk of cell carcinomas and adenocarcinomas. DNA methylation is one of the risk factors of cancer, and one‐carbon metabolism (OCM) played an important role in providing a methyl group. OCM involved many factors like vitamin B12, Hcy, folate, vitamin B6, and methionine. Decrease and accumulation of even one substance affected DNA methylation, genomic material, and tumor suppressor genes which lead to malignant transformation. In another study, the analysis showed elevated Hcy contributed to cancer (Yang, Li, Deng, & Wang, 2018).

Zhang, Wen, Wu, Guo, and Cui (2015) performed a meta‐analysis to examine the relationship between Hcy and folate with cancer and concluded that folate deficiency and increased serum Hcy associated with overall risk of cancer. Ovarian cancer also has a connection to the level of Hcy. Cancer cells derived from ovarian cancer patients had a deficit in the ability to remethylate Hcy. This changed metabolic condition results in elevated levels of serum Hcy (Kim, Kim, Roh, & Kwon, 2018).

### 4.15 Digestive tract cancer

Digestive tract cancer is the malignant gastrointestinal disease that covers gastric, esophageal, and colorectal cancer. Digestive tract cancer constitutes 22% deaths due to cancer every year, and malignancies of these cancers may be linked to hyper‐methylation and hypo‐methylation. If we find out the substance that affects cellular methylation, then digestive tract cancer diagnoses in early stage and several lives can be safe. Studies showed that an increase in Hcy caused elevation in S‐adenosylhomocysteine (SAH) which is a by‐product of remethylation reaction. Elevation in SAH results in hypo‐methylation of lymphocyte DNA. So, an indirectly elevated level of Hcy affects cellular methylation. Dose–response analysis also showed that 5 μmol/L increase in the concentration of Hcy enhanced 7% risk of digestive cancer (Xu et al., 2018). Several studies linked Hcy relation with digestive tract cancer like, and Wang et al. (2007) observed that people having a high concentration of Hcy than those having low showed an increased occurrence of gastric cancer means elevated Hcy increased risk of digestive tract cancer. One meta‐analysis demonstrated the relationship between blood tHcy and prostate cancer (Collin et al., 2010). Another meta‐analysis found that higher tHcy levels increased gastric cancer risk (Xu, Cheng, & Zhu, 2016).

### 4.16 Lung cancer

Lung cancer (LC) is also common and fatal cancer in the world. A meta‐analysis showed that serum Hcy was higher in LC patients than the serum level of Hcy in the control group. Further study demonstrated the difference of serum Hcy level in normal and LC patients while vitamin B6, folate level observed low than normal in these patients. This analysis showed that in OCM, vitamin B6 and folate act as a protective factor while Hcy contributes to lung cancer and can be used as a biomarker (Yang et al., 2018).

### 4.17 Colorectal cancer

In the United States, colorectal cancer (CRC) is the 3rd most commonly diagnosed cancer and the leading cause of death in both males and females. Many meta‐analyses were focused on determining the effects of dietary folate, supplements, and fiber on cancer and indicated that increased CRC risk is associated with increased tHcy and decreased folate level. The reason is that HHcy is responsible for oxidative stress and induction of the inflammatory responses, thus increasing the risk of CRC (Shiao et al., 2018). A study was conducted with postmenopausal women to assess the association between cysteine and Hcy with the incidence of CRC, and the result indicated that elevated plasma Hcy was associated with increased risk of CRC, whereas high cysteine level was associated with decreased risk. So HHcy low cysteine is risk factors for incident CRC (Miller et al., 2013). Patients with the inflammatory intestinal disease have a greater chance of developing colorectal cancer when their Hcy levels are higher than in healthy individuals (Keshteli, Baracos, & Madsen, 2015). Dietary factors and lifestyle may contribute to the increasing CRC incidence like obesity, fat diet, alcohol consumption, and smoking (Baena & Salinas, 2015). Fat diet is also considered as the main cause of CRC the reason is maybe that the high‐fat diet is responsible for increasing plasma Hcy levels in our body (Jakubowski, 2019).

### 4.18 Vitiligo

Hyperhomocysteinemia has been reported with vitiligo (multifactorial pigmentary disorder). Worldwide, the prevalence of this disease is around 1%. Its etiology may be due to the destruction of melanocytes, oxidative stress, and autoimmunity. Many studies have reported an elevated level of Hcy in vitiligo and concluded Hcy increased oxidative stress and disrupted melanocytes. A meta‐analysis reported a high level of Hcy and low levels of folate, vitamin B12, and vitamin B6 in the patients suffering from vitiligo as compared to the control group. Several hypotheses like inhibition of melanin synthesis enzyme (tyrosinase) or increased ROS production by Hcy can explain the elevated level of Hcy in vitiligo (Tsai, Kuo, & Huang, 2018).

### 4.19 Homocysteine and body composition

A study conducted on nine homocystinuria patients showed that reduced fat mass is common in these patients and alternation in choline and Hcy pathways affects lipid metabolism and body mass (Poloni et al., 2014). Yin et al. (2016) investigated the effect of L‐cysteine and found that L‐cysteine effectively reduces final body weight, body weight gain, food intake, and feed efficiency in rats.

## 5 CONCLUSION

Cysteine and Hcy are sulfur‐containing AAs. Hcy is an intermediate product of methionine conversion into cysteine. Cysteine and Hcy both have many important roles in the body, but Hcy is considered as toxic specially hyperhomocysteinemia condition which is associated with many medical problems. HHcy may be due to mutation in its metabolic pathways and folate, vitamin B12, and vitamin B6 deficiency. Hcy and Cys can be used as a biomarker of many diseases like CVD, neurological disorder, diabetes, cancer, vitiligo, and renal dysfunction because high Hcy and low cysteine level seen in these diseases but in CVD condition role of cysteine are not clear. Further research should be needed so that both cysteine and Hcy can be used clinically on a large scale for future uses.

## CONFLICT OF INTEREST

The authors declare no conflicts of interest.
